# Watching single molecules in action

**DOI:** 10.7554/eLife.02061

**Published:** 2014-01-28

**Authors:** Jordan Monnet, Terence R Strick

**Affiliations:** 1 **Jordan Monnet** is in the Institut Jacques Monod, CNRS UMR 7592, Université Paris Diderot, Paris, Francemonnet.jordan@ijm.univ-paris-diderot.fr; 2 **Terence R Strick** is in the Institut Jacques Monod, CNRS UMR 7592, Université Paris Diderot, Paris, Francestrick.terence@ijm.univ-paris-diderot.fr

**Keywords:** single-molecule, real-time, transcription, fluorescence, in situ hybridization, unstructured nucleic acid, *E. coli*, Viruses

## Abstract

A fluorescent imaging technique called fastFISH has been used to track the various steps involved in the transcription of a single DNA molecule.

**Related research article** Zhang Z, Revyakin A, Grimm JB, Lavis LD, Tjian R. 2014. Single-molecule tracking of the transcription cycle by sub-second RNA detection. *eLife* **3**:e01775. doi: 10.7554/eLife.01775

**Image** 357 fastFISH images (taken 0.4 seconds apart) showing four representative transcription events

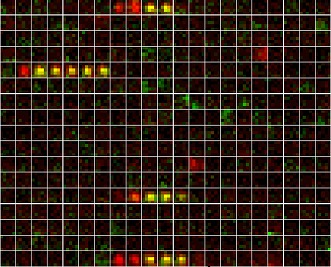

Single-molecule techniques allow researchers to follow, in real-time, the details of biochemical reactions that are obscured when experiments are carried out on a large number of molecules (Kapanidis and Strick, 2009). These details include transient molecular species and the kinetics of molecular complexes. For example, the ability to observe both the assembly and disassembly of molecular complexes, rather than the equilibrium between these two process, could provide mechanistic insights into the way that these complexes perform various functions in cells. Moreover, if one of the molecules involved in a biochemical reaction can be tethered to a solid support, such as a glass coverslip, and placed in a solution, the effects of random diffusion can also be eliminated: this will make it possible to continuously monitor how the tethered molecule interacts with other molecules in solution.

The future of the field lies in techniques that can directly and simultaneously monitor an ever-expanding number of reagents with ever-increasing throughput and ever-better time resolution (Friedman et al., 2006; Uemura et al., 2010; Hoskins et al., 2011; Revyakin et al., 2012; Chen et al., 2014). Now, in *eLife*, Zhengjian Zhang, Andrey Revyakin and co-workers have applied these principles to capture the full reaction cycle of a simple yet highly kinetic system, the T7 RNA polymerase (RNAP), in unprecedented detail (Zhang et al., 2014). T7 RNAP is an enzyme that binds to a stretch of DNA called the T7 promoter and starts the process by which downstream genes are transcribed to produce RNA molecules (which are subsequently translated to produce proteins).

Although the multi-step process of transcription has been studied extensively, the fast kinetics of T7 RNAP present unique challenges, and these have made it difficult to study the workings of this enzyme at single-molecule resolution (Skinner et al., 2004; Thomen et al., 2008; Skinner et al., 2011). Indeed, T7 RNAP is capable of initiating transcription and escaping from the promoter (an important part of the transcription process) within a second of binding to the promoter. It can also transcribe several hundreds of nucleotides per second. Therefore, in order to assess the full transcription cycle of T7 RNAP, it is essential to rapidly and directly monitor two processes: the binding of RNAP to DNA, and the synthesis of RNA. Until now, however, techniques for the direct detection of the nascent RNA have been too slow to follow the fast kinetics of T7 RNAP.

Zhang, Revyakin and co-workers—who are based at the Janelia Farm Research Campus and the University of California Berkeley—developed a method that allows them to directly and independently monitor these two processes. The binding of RNAP to DNA was monitored by adding a fluorescent label (a Cy5 molecule) to the RNAP, and the nascent RNA was monitored with a fluorescent DNA probe that consists of a single strand of DNA attached to a fluorescent Cy3 molecule (Figure 1). The technique used to probe for the nascent RNA is called fastFISH because it is a faster version of an existing technique called fluorescence in situ hybridization (FISH).

**Figure 1.**
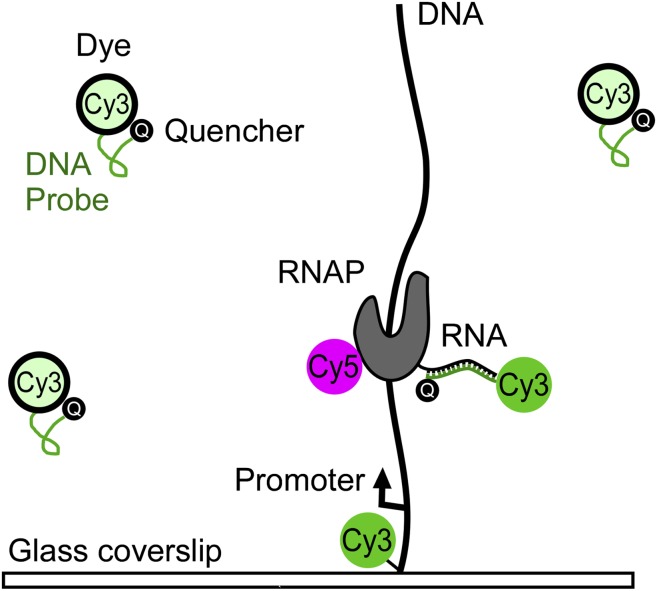
Tracking the transcription of a DNA molecule with the fastFISH method. The DNA molecule is labelled with a fluorescent Cy3 molecule and tethered to a glass cover slip; an optical field (not shown) is then used to generate fluorescence, which allows the position of the DNA molecule to be determined. Next, the Cy3 label on the DNA molecule is bleached out so Cy3 can be used to label the DNA probe. The T7 RNA polymerase (RNAP), which is labelled with a fluorescent Cy5 molecule, starts to transcribe the DNA molecule to produce an RNA molecule, and the Cy3 molecule in the DNA probe starts to fluoresce when the probe binds to the nascent RNA molecule. The RNAP and the DNA probe can be monitored independently and simultaneously (with a time resolution of 80 milliseconds) because the Cy5 and Cy3 molecules fluoresce at different wavelengths. It is also possible to distinguish between T7 RNAP bound to a promoter on the DNA and T7 RNAP transcribing the DNA because, in the latter case, the fluorescence from the Cy5 will coincide with fluorescence from the Cy3.

FastFISH is based on two key ideas: first, in order to achieve high rates of hybridization between the DNA probe and the nascent RNA, the probe must not form stable secondary structures. This is achieved by using only three of the four nucleotides found in DNA—adenine, thymine and guanine—to design the DNA probe, and results in hybridization rates that are close to the maximum possible rate allowed by diffusion. However, relatively high concentrations of the DNA probe are needed to achieve a time resolution of better than 1 second, and this causes a problem: the high levels of background fluorescence from the DNA probes will overwhelm the fluorescence from the single molecules we want to study. Zhang et al. overcome this problem with a second key idea—they attach a quencher molecule to their DNA probe to switch off the fluorescence of the Cy3 molecule. However, the fluorescence is switched on when the DNA probes binds to the nascent RNA. By eliminating background fluorescence, these ‘self-quenching’ DNA probes are able to detect nascent RNA with a time resolution on the order of 80 milliseconds.

Using this approach, Zhang et al. measured the key kinetic features of the T7 RNAP transcription cycle. The binding of T7 RNAP to promoter DNA is very fast, limited only by diffusion, but this binding does not always result in transcription because the RNAP can also dissociate from the DNA. However T7 RNAP is biased towards productive transcription, and is capable of initiating transcription and carrying out promoter escape within 0.2 seconds of binding to the promoter.

By demonstrating the ability to directly monitor multiple components of the viral T7 transcription machinery in real time with a resolution of better than one second, Zhang, Revyakin and co-workers show how single-molecule experimentation is rapidly evolving into multi-molecule experimentation. This occurs at a key moment in biochemistry: ever-larger macromolecular complexes can routinely be purified and analysed structurally (Gonen et al., 2012), but performing detailed kinetic analysis on these complexes remains challenging. By developing ways to monitor multiple components simultaneously in real-time, and by providing ways to work in near-physiological conditions (Hoskins et al., 2011), single-molecule experimentation will continue to provide new tools to help narrow the gap between biochemistry carried out in vitro and in vivo.
